# Validation of knowledge‐based and multicriterial optimization assistive auto‐planning algorithms for prostate VMAT radiotherapy using biological optimization

**DOI:** 10.1002/acm2.70334

**Published:** 2025-11-05

**Authors:** Willem P. E. Boonzaier, Lourens J. Strauss

**Affiliations:** ^1^ Department of Medical Physics University of the Free State: School of Medicine Bloemfontein South Africa

**Keywords:** Auto‐planning, Knowledge‐based planning, Monaco scripting, Multicriterial optimization

## Abstract

**Purpose:**

External beam radiotherapy for cancer treatment historically has faced the challenge of delivering sufficient dose to the target while minimizing dose to critical organs. Inverse planning techniques in modulated therapy can improve organ‐at‐risk (OAR) sparing but require significant human resources and can depend on planner experience. Advances in software and artificial intelligence (AI) have enabled the development of commercial treatment planning systems (TPS) with scripting and auto‐planning capabilities, potentially reducing human resource demands and standardizing plan quality. This study aimed to create and validate knowledge‐based planning (KBP) and multicriterial optimization (MCO) algorithms for assistive auto‐planning of prostate volumetric modulated arc therapy (VMAT) plans using the Elekta Monaco TPS.

**Methods:**

Our methodology involved implementing and validating these algorithms retrospectively. We compared dose volume histogram (DVH), generalize equivalent uniform dose (gEUD), and plan quality scores with KBP and MCO algorithms to the clinical plans.

**Results:**

KBP and MCO generated plans on average spared OARs better and on average had better conformity to the targets whilst not sacrificing target coverage. Additionally, algorithm generated plans could be produced within 30 min and were of clinical quality for 72% (KBP) and 78% (MCO) of plans. When comparing the KBP and MCO plans, MCO was dosimetrically slightly superior, slightly faster, and produced plans of clinical quality in more of the validation population than the KBP algorithm.

**Conclusion:**

This work showed that it is possible to build KBP and MCO based assistive auto‐planning in the Elekta Monaco TPS focusing on gEUD based cost functions. When using the Monaco Scripting functionality, these algorithms could assist in reducing the clinical planning workload while maintaining a patient specific standard of quality.

## 1 INTRODUCTION

External beam radiotherapy as a treatment modality for cancer patients has the challenge of achieving sufficient target dose while minimizing the dose inevitably also delivered in critical organs. Inverse planning techniques applied in modulated therapy can improve sparing of organs‐at‐risk (OARs) while maintaining target coverage. However, the disadvantages of inverse planning techniques include increased human resource time for planning compared to conventional techniques, and plan quality dependence on planner experience.^1^ Recent advances in software development and artificial intelligence (AI) have led to commercial treatment planning software companies giving access to some features of the treatment planning system (TPS) via scripting or commercial auto‐planning software packages. This provides an opportunity to replace mundane or systematic tasks performed by users to be coded, which has the potential to alleviate the need for extensive human resources and at the same time provide a standard of quality that is optimized for the patient`s anatomy.^2^

One such approach is knowledge‐based planning (KBP), where a model is built from knowledge of previous acceptable plans to predict outcomes for current plans, and remains one the most robust and clinically used auto‐planning methods. A clinical example is Varian's KBP algorithm, RapidPlan, which utilizes a trained model that predicts dosimetric outcome from OAR proximity to the target volume and inclusion in the Beam's‐eye view (BEV) from a known population of plans.^3^

The robustness of the RapidPlan algorithm was demonstrated by Chung et al. showing that KBP algorithms can be used to create high quality, consistent, clinically acceptable plans efficiently with no user input for 10 different treatment sites. The authors claim that KBP algorithms have the potential to significantly impact treatment planning workflows when implemented appropriately.^4^

Nakamura et al. published work on training successive RapidPlan models to improve plan quality in a stepwise method over time. Notably, the authors found that when improving the input plans when training the model, plan quality had improved, and regression scatterplots had become more converged showing a better overall agreement in quality of input plans.^5^

Additionally, many modern radiotherapy TPSs incorporate a pareto based multicriterial optimization (MCO) algorithm. This function in the Elekta Monaco TPS is designed to exceed the constraint criteria for OARs while target coverage is maintained.^6^ Tonneau et al. showed that by implementing an MCO function in the Raystation TPS for head‐and‐neck VMAT plans in the optimization phase, a reproducible sparing of OARs could be achieved improving consistency and possibly patient quality of life.^7^

Carlos et al. showed the effect on plan quality when optimizing with RapidPlan and MCO algorithms. When considering prostate patients who had received treatment to the prostate and nodes, the MCO algorithm produced on average plan quality scores which were only slightly better than when using RapidPlan.^8^

Prior efforts have been made to automate optimization workflows in the Monaco TPS, but most systems made use of customer generated infrastructure to do so since the Monaco scripting interface was not yet available at the time. These algorithms generally used rule‐based approaches to mimic exploratory processes that planners would perform when planning.^9^, ^10^ Other studies have shown the effectiveness of using the MCO algorithm when applied sequentially according to a predefined user‐assisted order of priority. The interface through which this was performed was not explicitly stipulated.^11^ To our knowledge, no KBP algorithm or any predictive based models exists in the Monaco TPS for clinical use; however, a MCO function is available which can be activated per cost function. This function has predefined settings that cannot be adjusted.

The Monaco TPS additionally allows by default the use of biological optimization parameters with equivalent uniform dose (EUD) as a planning parameter, which is not a standard option in all TPSs. The definition of the generalized equivalent uniform dose (gEUD) by Niemierko is the “uniform dose that, if delivered over the same number of fractions as the non‐uniform dose distribution of interest, yields the same radiobiological effect”.^12^ This report defines a tissue‐specific parameter a that describes the volume effect. For a→−∞, the gEUD approaches the minimum dose; therefore, minimum values for parameter a can be used to force dose into a structure, as in the case for targets. When a→+∞, gEUD approaches the maximum dose and for a=1, the gEUD is equal to the mean dose. Therefore, positive values of parameter a can be used to control dose in any OAR from a mean dose to a maximum dose.^12^

Some studies have reported that the use of dose volume (DV)‐based cost functions often lead to satisfying several local minima in the optimization problem, which may not be the best global solution to the problem. Between 2002 and 2007, many studies demonstrated the advantages of introducing EUD‐based cost functions into the inverse optimizer for modulated treatment planning. The consensus was that when introducing EUD‐based cost functions, OAR sparing could be improved while maintaining target coverage. Furthermore, the combination of EUD‐based and DV‐based cost functions presented a robust means of obtaining the desired dose distribution.^13^, ^14^, ^15^, ^16^, ^17^, ^18^, ^19^

To bridge these gaps, the American Association for Physics in Medicine (AAPM) in 2012 published a report of task group 166 giving recommendations regarding the use and QA of biological functions in treatment planning. This report in general recommends that EUD based cost functions should be used in conjunction with maximum dose‐type cost functions per structure to deliver appropriate clinical plans and plan evaluation should be performed based on DV criteria in combination with a review of the 3D dose distribution. Additionally, plan ranking may be done using EUD if it is calibrated appropriately.^20^

Another important consideration is dose delivery accuracy, especially since the complexity of modulated treatments introduces factors that can affect both plan quality and deliverability. This is verified during pretreatment quality assurance (QA) using phantom measurements.^21^ Such verification is particularly important when producing auto‐assisted plans since these plans also alter the contribution of small fields and rapidly moving components like multileaf collimators (MLCs).^22^, ^23^ Small field segments pose challenges due to dosimetric uncertainties arising from detector resolution limitations and loss of lateral charged particle equilibrium.^24^ Similarly, high modulation and fast MLC motion can increase the risk of delivery errors, making robust QA processes critical.^25^

## 2 PURPOSE

The aim of this work was to create and validate both a knowledge‐based and multicriterial optimization algorithm for assistive auto‐planning in our clinic for prostate VMAT plans within the Elekta Monaco TPS driven by EUD‐based cost functions.

## 3 MATERIALS AND METHODS

### 3.1 Patient selection and planning

Prostate cancer patients treated in 2023 with staging T1–T3 were included in this study. Patients that were not planned using the standard departmental protocol were excluded. Thirty‐six (36) consecutive prostate cancer patients were selected of which the first eighteen (18) patients were defined as the training population and the second eighteen (18) as the validation population.

The departmental hypo‐fractionated treatment protocol was followed for all cases delivering 60 and 45 Gy to the primary and nodal planning target volumes (PTVs), respectively, over 20 fractions. Clinical plans consisted of a dual‐arc VMAT technique using 10MV. Plan quality was assessed considering gEUD and DVH criteria to the PTVs and OARs, as summarized in Table 1. For clinical acceptability, the DVH criteria were required to be met and a visual inspection of the dose distribution by the radiation oncologist performed. These values are standard in our clinic based on both ICRU and QUANTEC.^26^, ^27^ A clinical decision superseding this protocol could sometimes be made by the radiation oncologist when target coverage and OAR sparing could not be both satisfied.

**TABLE 1:** Constraints for clinical plan acceptability.

	Constraints	
Structure	Volume	Dose (Gy)
PTV 60 Gy	> 98%	58.6
	< 2 %	62.9
PTV 45 Gy	> 98%	43.6
Bladder	< 15%	64.3
	< 25%	61.7
	< 35%	58.9
	< 50%	56.0
Rectum	< 15%	61.7
	< 20%	58.9
	< 25%	56.0
	< 30%	54.3
	< 35%	53.1
	< 50%	46.8
Bowel Bag	Max	46.8
	< 195 cm^3^	43.5

The training population was used to train the KBP algorithm. The validation population was then retrospectively used to validate both the KBP and MCO algorithms against the original clinically accepted plans. All plans were created using the Elekta Monaco (V6.1.4.0) TPS. Both algorithms were created using the Monaco scripting Application Programming Interface (API) interfacing with Monaco V6.1.4.0 to eliminate user variability.

**FIGURE 1:**
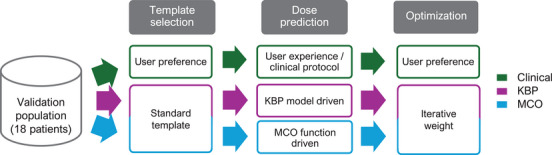
Method overview of different algorithms, namely clinical, knowledge‐based planning (KBP), and multicriterial optimization (MCO).

An overview of the study flow is given in Figure 1 with the details described in the following sections.

#### 3.1.1 Optimization parameters

Clinical plans were optimized utilizing mostly EUD‐driven cost functions in the constrained mode of optimization, and dose calculation using Monte Carlo. Five qualified medical physicists with varying levels of experience produced the clinical plans.

Algorithm generated plans were all produced from a single plan template using identical cost‐function sets as specified in Table 2. The same number of arcs, maximum number of control points, segmentation settings, optimization mode, and dose calculation algorithm used for clinical plans were adopted for the algorithm generated plans as well. All target and OAR contours were used as optimization structures. Target EUD, Target penalty and Quadratic overdose cost functions were used to drive uniformity in the targets while Quadratic overdose functions were used to control the maximum dose to each OAR. Serial cost functions were used to control the dose in overlapping and nontarget‐overlapping parts of each of the OARs. Additionally, three Quadratic overdose functions with varying shrink margins were used to control the dose fall‐off around the targets, based on distance from the respective PTVs. Note the parameters, indicated in bold, which the algorithm was allowed to modify in Table 2.

**TABLE 2:** Standard template with initial cost‐functions and parameters for planning in TPS.

Structure	Cost function	Reference dose (Gy)	Iso‐constraint (Gy)	Margin (cm)
CTVs 60 Gy	Target EUD	–	61.50	–
CTVs 45 Gy	Target EUD	–	48.50	–
PTV 60 Gy	Target penalty	–	59.00	–
	Target EUD	–	61.00	–
	Quadratic overdose	62.00	0.20	–
PTV 45 Gy	Target penalty	–	45.00	–
	Target EUD	–	45.00	0.6^*^
	Quadratic overdose	51.00	0.20	–
Bladder	Serial (*k* = 8)	–	**53.10**	–
	Quadratic overdose	61.50	0.05	–
Bladder (non‐overlap)	Serial (*k *= 4)	–	**45.50**	–
Rectum	Serial (*k* = 12)	–	**55.40**	–
	Quadratic overdose	61.50	0.05	–
Rectum (non‐overlap)	Serial (*k* = 8)	–	**45.50**	–
Bowel Bag	Serial (*k* = 12)	–	**47.50**	–
	Quadratic overdose	**45.00**	0.05	–
Patient / unspecified	Quadratic overdose	**58.00**	0.05	0.2^*^
		**43.00**	0.15	1.0^*^, 0.3^**^
		**38.00**	0.15	1.5^*^, 1.0^**^

* Distance from PTV 60 Gy.

** Distance from PTV 45 Gy.

Values in bold indicate parameters which the algorithm may modify.

#### 3.1.2 Algorithm configuration

Since planners used their personal preference for cost functions in the clinical plans, variation in the number of gEUD cost functions and the power law exponent used per OAR posed difficulty in correlating planning gEUDs with structure overlap. To overcome this, planners agreed on a standard set of cost functions, as mentioned in Table 2, with which all clinical plans were re‐optimized as a means of data augmentation. This introduced some bias to the standard set of cost functions and therefore algorithms were only allowed to spare OARs to a point where it did not negatively affect PTV coverage. Thus, a standard optimization approach was introduced which needed to be validated by a planner on a patient‐by‐patient basis, giving autonomy to the planner. These plans were only used for training of the KBP plans. No comparison was made to these plans, but rather to the radiation oncologist's clinically accepted plans.

During the modelling phase for the KBP algorithm, regression analysis was performed between the fractional overlap of each OAR with each PTV (using R Version 4.4.2, The R Foundation for Statistical Computing). Cost functions that had the best correlation with anatomical overlap of each OAR were modelled linearly to create the knowledge‐based portion of the plan prediction. Fractional overlap for each OAR with each PTV was derived from the dice similarity coefficient obtainable in the structure analysis workspace of the Monaco TPS. These fractional overlap measures were then used as input to the regression models created to predict a starting gEUD for optimization.

For development of the MCO algorithm, the standard template of cost functions was used as starting point, with the cost functions that showed strong correlation with anatomical overlap in the KBP algorithm phase enabled for multicriterial optimization in stage 1 of optimization.

Since optimization can be summarized as the balance between coverage of the targets and lowering dose to each OAR, the two algorithms were designed in two steps. The first step in each algorithm focused on lowering the dose to each OAR to what each of the algorithms predicted the best sparing would be, even if it meant compromising target coverage slightly. Then, an additional iterative logistic regression algorithm was employed in stage 1 of optimization in both approaches to ensure that sufficient target coverage was regained. Here, the Lagrange multiplier generated cost function weight was utilized as input to the logistic regression algorithm to determine if a cost function should be adjusted to regain target coverage after optimizing OAR sparing. Simply put, in an iterative manner, the Monaco scripting interface was allowed to adjust the gEUD or quadratic overdose functions of the OARs until all Lagrange multiplier generated cost function weights were lower for the OAR than the PTVs.

### 3.2 Testing and validation

Validation of the algorithm generated plans against the clinical plans were done by comparing the plan quality using gEUDs as defined by Niemerko et al.^19^, ^28^, ^29^ For the gEUD analysis, power law exponents of−10, 12 and 8 were used for the targets, rectum and bladder volumes, respectively. When determining the gEUD for the bowel bag, a power law exponent of 4 was applied to the 200 cm^3^ of the bowel bag that received the highest dose.^30^

Algorithm generated plans were evaluated quantitatively for clinical acceptability in the same manner as the clinical plans, as stated before using Table 1.

Conformity indices (CI) as recommended in the ICRU 83 publication were calculated from the ratio of the physical volume V receiving 95% of the prescribed dose to the volume of the corresponding PTV, as shown in Equation (1).^31^

(1) $$CI=\frac{V_{95\%}}{V_{PTV}}$$

From the same publication, the homogeneity indices (HI) in terms of the dose D to different volumes were calculated as given in Equation 2 below:

(2) $$HI=\frac{\left(D_{2\%}-D_{98\%}\right)}{D_{50\%}}$$

Significant differences in plan quality indicators and gEUDs for clinical versus algorithm‐generated plans were calculated using a Wilcoxon signed rank test. All statistical analyses were performed using R and a *p *< 0.05 was considered statistically significant. Additionally, multiple comparison correction using the Benjamini–Hochberg method was applied to limit the probability of false positives in the small study sample. A false discovery rate of 5% was used for this purpose.

### 3.3 Practical considerations and quality assurance

Some aspects of the practicality of each algorithm were evaluated by considering the total time taken to reach a deliverable plan as well as the success rate of achieving a clinically acceptable solution. Total planning time was coded into the planning script by calculating the time difference between the start and the completion of the planning script.

Each plan was delivered on an Elekta Versa HD linear accelerator and patient specific QA was performed according to the departmental protocol. This involved fluence measurements of each plan with the IBA Dolphin transmission detector and subsequent reconstruction into the planned patient anatomy using the IBA Compass software. Gamma(Γ) pass rates for the total patient volume, PTV 60 Gy and PTV 45 Gy regions of interest using a gamma criterion of 3 mm/2%, as well as the total delivery time and monitor units were recorded for each plan. These metrics were compared to clinical plans by means of Wilcoxon signed rank test. All statistical analyses were performed using R and a *p *< 0.05 was considered statistically significant.

## 4 RESULTS

### 4.1 KBP model configuration

**FIGURE 2:**
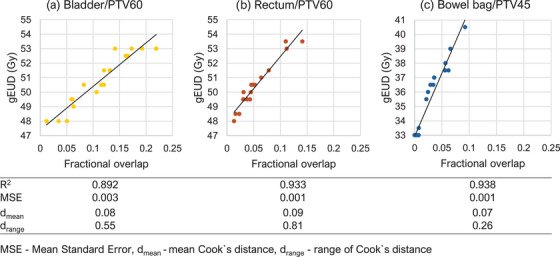
Correlation between OAR/target overlap and achieved gEUD (*n* = 18) for each OAR/target combination, with analysis metrics shown at the bottom.

A very strong positive linear correlation between OAR overlap with PTV 60 Gy could be seen with the gEUD of the rectum and bladder, while an even stronger positive linear correlation was observed between OAR overlap with PTV 45 Gy and the bowel bag gEUD. This is illustrated graphically in Figure 2 with the R^2^, mean standard error (MSE), as well as the mean and range of the Cook`s distance d for each fit shown at the bottom. The bladder shows the largest MSE of 0.003 while the largest average Cook`s distance and Cook`s distance range was observed for the Rectum.

### 4.2 Testing and validation

**FIGURE 3:**
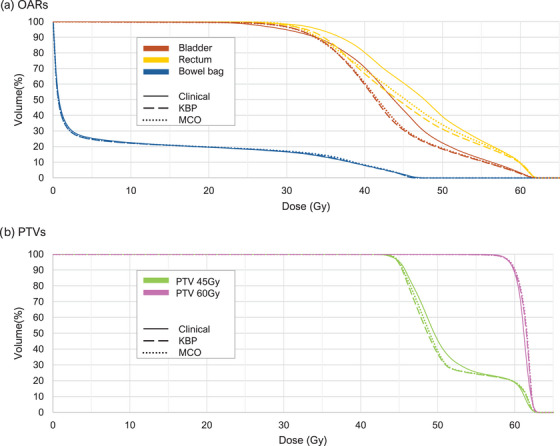
Average DVHs per algorithm for (a) OARs and (b) PTVs when comparing planning methods (*n* = 18).

Figure 3 shows average DVH comparisons of a) the OARs and b) PTVs in the clinical, KBP and MCO plan populations to assess the average plan quality for each technique. Additionally, Table 3 shows a summary of the mean gEUD as well as average CI and HI calculated for each planning method. Table 3 also explores the statistical significance of the observed differences via *p*‐values.

**TABLE 3:** Comparison of average gEUD and plan quality indices across planning methods.

		Mean ± SD (Gy)			*p*‐value		
		Clinical	KBP	MCO	Clinical vs. KBP	Clinical vs. MCO	KBP vs. MCO
gEUD	PTV 60 Gy	61.0 ± 0.2	61.2 ± 0.2	61.2 ± 0.3	0.96	0.99	0.82
	PTV 45 Gy	49.3 ± 1.4	48.6 ± 1.0	48.8 ± 1.0	**0.03**	**0.02**	0.78
	Bladder	51.8 ± 1.8	50.7 ± 2.5	50.9 ± 2.9	**0.01** ^*^	0.06	0.98
	Rectum	50.6 ± 1.4	49.8 ± 1.4	49.8 ± 1.9	0.05	**0.01** ^*^	0.57
	Bowel bag	41.7 ± 3.5	40.5 ± 4.8	41.0 ± 4.6	0.29	**0.04**	0.60
Indices	CI PTV60	1.2 ± 0.1	1.1 ± 0.1	1.1 ± 0.1	**0.01** ^*^	0.20	1.00
	CI PTV45	1.8 ± 0.4	1.5 ± 0.1	1.5 ± 0.1	**0.01** ^*^	**0.01** ^*^	1.00
	HI PTV60	0.1 ± 0.0	0.1 ± 0.0	0.1 ± 0.0	0.94	0.73	0.25

*Notes*: p‐values in bold are statistically significant.

Abbreviations: CI—Conformity Index, HI—Heterogeneity Index, KBP—Knowledge‐based planning, MCO—Multicriterial optimization.

* Statistically significant even after Benjamini–Hochberg correction.

When comparing the dose to the targets for both algorithms to the clinical plans, the PTV 60 Gy differences were not statistically significant, however a statistically significant decrease to the gEUD to PTV 45 Gy was noted for both algorithms when comparing to the clinical plans. The KBP algorithm had a statistically significant improvement to the gEUD of the bladder, and the MCO algorithm a statistically significant improvements to the gEUD of the rectum and the bowel bag. Additionally, the KBP algorithm showed to have a statistically significant improvement in conformity to PTV 60 Gy, while both algorithms were statistically significantly better in conforming to PTV 45 Gy, potentially saving dose to non‐contoured normal tissue. No statistically significant differences in homogeneity in PTV 60 Gy were observed.

No statistically significant differences were observed when comparing gEUD and plan quality indices of the two algorithm generated plan populations, although slightly better clinical quality was observed using the MCO algorithm.

### 4.3 Practical considerations and quality assurance

**FIGURE 4:**
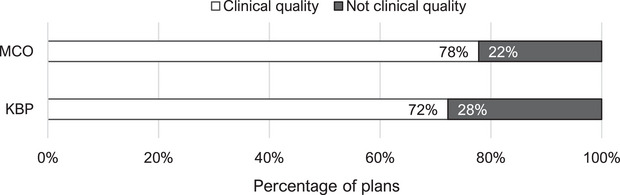
Success rate per planning technique (*n* = 18).

The average time required to reach a clinically acceptable plan was 27 min and 18 s for KBP and 25 min and 12 s for MCO scripted planning techniques. Additionally, the MCO algorithm showed a slightly higher rate (78%) of producing clinically acceptable plans than the KBP algorithm (72%), as can be seen in Figure 4.

Table 4 below shows a summary of the statistical testing of the quality assurance parameters.

**TABLE 4:** Comparison of quality assurance parameters across planning methods.

	Mean ± SD (%)			*p*‐value	
Metric	Clinical	KBP	MCO	Clinical vs KBP	Clinical vs MCO
Γ pass rate: Patient	99.3 ± 0.3	99.3 ± 0.4	99.3 ± 0.4	0.66	0.68
Γ pass rate: PTV 60 Gy	96.3 ± 4.4	97.1 ± 3.4	97.6 ± 2.3	0.79	0.81
Γ pass rate: PTV 45 Gy	98.0 ± 1.2	98.8 ± 1.1	98.5 ± 1.2	0.98	0.87
Delivery Time (min)	2.7 ± 0.4	3.0 ± 0.3	3.0 ± 0.2	0.96	0.97
Total MUs	1034.0 ± 149.8	1174.0 ± 162.9	1178.2 ± 108.4	0.96	0.98

*Notes*: Gamma(Γ) pass rates are for 3 mm/2% criteria.

Abbreviations: KBP—Knowledge‐based planning, MCO—Multicriterial optimization.

The gamma pass rates over all plans were consistently high in both clinical and algorithm generated plans. Both scripted planning techniques generated plans that took on average 18 s longer to deliver. On average the scripted plans had slightly higher total MUs with an average difference from clinical plans of 140 and 144 MUs for KBP and MCO respectively. When comparing delivery time and MUs of scripted planning techniques to that of clinical plans, no statistical differences were observed.

## 5 DISCUSSION

### 5.1 Model configuration

The R^2^ values show high correlation between fractional overlap of OARs and targets. Additionally, the MSE and mean Cook`s distance (d_mean_) observed in this study are much lower than the values reported by Nakamura et al.,^32^ showing much less outliers in our training data. This could be due to a variety of reasons, including the difference in delineation in the OAR between the studies (e.g., bladder vs. bladder wall), the difference in the decision of the dosimetric parameter being modelled (EUD vs. geometric equivalent DVH), and the lower number of plans included in the population for this study.

### 5.2 Testing and validation

When considering the DVH for PTV 45 Gy, the coverage of both algorithms compared well with the clinical plans, although a steeper DVH slope beyond the shoulder can be observed, indicating better uniformity in this PTV for algorithm generated plans. This change in DVH will alter the gEUD, leading to the significant differences observed. The lower observed gEUD for PTV 45 Gy therefore does not indicate a drop in coverage to this PTV but rather an improvement of homogeneity within this volume in algorithm generated plans.

The larger standard deviation for bladder and rectum gEUDs in the MCO plans compared to the KBP plans alludes to better sparing in cases where more sparing is possible. This agrees with published results from Carlos et al, showing a mild improvement in overall plan quality scores when using a MCO algorithm compared to a KBP algorithm.^8^ The consistency in heterogeneity of the PTV 60 Gy on the other hand linked with meeting the minimum and maximum dose limits set confirms the effectiveness of the methods employed in the second step of optimization to recover target dose.

When modelling the KBP algorithm, the correlation was very high for all OARs, which presented overfitting of the training data population as a possible concern. Validation was performed on an independent population to rule out overfitting to the training data. The data shows that overfitting could be avoided by allowing the secondary logistic regression algorithm to adjust predicted gEUDs to ensure appropriateness on a validation population.

In addition to matching clinical plan quality, both algorithms were on average able to improve OAR sparing and conformity whilst keeping target coverage at an acceptable level.

### 5.3 Practical considerations and quality assurance

Both algorithms showed a quick optimization time on average of less than 30 min, producing clinically acceptable plans in more than two thirds of the cases. The MCO algorithm was on average 2 min faster than the KBP algorithm.

The MCO algorithm also had a slightly higher chance of creating clinically acceptable plans when compared to the KBP algorithm. These differences are small and either algorithm would be a reasonable solution in a clinical setting. However, it should be noted that due to the differences in mechanisms the KBP method predicts dosimetric outcome based on a trained model and will only be as diverse as the anatomy included in the training population, whereas the MCO algorithm adapts on a patient‐by‐patient basis. For patients with atypical anatomies compared to that in the training population it would therefore make more sense to use a MCO approach to predict dosimetric outcomes.

A direct time comparison to the clinical planning time was not feasible in this study due to the retrospective nature of the work. Planning time was not routinely documented for the clinical plans at the time they were created, and thus objective data was unavailable for comparison. Additionally, clinical planning was performed by planners who were simultaneously engaged in other responsibilities, including clinical medical physics support and teaching activities. Because of this, the actual time spent on planning was fragmented and did not reflect continuous effort, making it challenging to determine an accurate planning duration. However, based on departmental standards, a turnaround time of approximately one working day per plan is generally expected in clinical practice. Knowing this, it is important to highlight that the automated process, even in the worst‐case scenario where no clinically acceptable plan is generated automatically, would only extend the total planning time by approximately 30 min. In most cases, we expect that the planner could continue planning from the auto‐generated result, which provides a structured starting point thereby reducing the total planning time.

Even though scripted planning techniques generated more MUs and took slightly longer to deliver on average, these increases were marginal with no real clinical impact. This contrasts sharply with the clear improvements in plan quality seen in the scripted plans, suggesting that while user‐generated plans may generally meet clinical acceptability, they may not always represent the optimal treatment plan for the patient.

## 6 CONCLUSION

This study aimed to create and validate both knowledge‐based and multicriterial optimization algorithms for assisted auto‐planning in our clinic for prostate VMAT plans using the Monaco TPS with EUD‐based cost functions. The results show that it is possible through Monaco scripting and can provide good quality clinical plans. Additionally, using the multicriterial option within the Monaco TPS gives the best possible OAR sparing for most cases.

When using Monaco scripting to officiate the presented algorithms, a good quality plan can be produced without user intervention in a short period of time and is clinically usable as is in many cases. Plans which are not of clinically acceptable quality would then require minimal user input additionally to achieve a usable plan. Additional to matching clinical quality in most plans, variation in plan quality due to what we assume to be user preferences or experience could therefore also be minimized by providing a high‐quality starting point on which the planner might improve on. Practical considerations also showed that the increase in consistency and quality does not necessarily come at a significant time or quality cost. This therefore has the possibility to reduce planning workload as an assistive tool while increasing overall plan quality in the clinic.

When comparing our approach to other published work, we also observed more consistency and on average lower OAR doses when using these assisted auto‐planning approaches, although most other studies make use of TPS solutions from other vendors. To our knowledge this is the first published work on a knowledge‐based assistive auto‐planning algorithm in the Monaco TPS that uses gEUD as a dose predictor.

Designing and implementing assistive auto‐planning in any clinic using the methodology as described in this study would only require experience with the Monaco scripting tool, as well as development of the models from the clinic's own population and planning protocols. Caution should however be exercised as any assumption made in the process could influence a clinical endpoint in the entire patient population. Emphasis should be placed on independent review by qualified personnel. When adopting new planning techniques, it is often recommended that double planning is done for a trial period before transitioning to a new workflow completely.^20^

Some limitations in this study include timing of the clinical non‐automated plans to enable a true comparison in terms of timesaving, as well as quantification of the time and effort required to get to clinically acceptable plans in the cases where the auto‐assisted plans were not yet adequate.

Future research includes expanding this work to include other treatment sites such as head‐and‐neck, evaluating planner feedback to the model to assist in improving optimization techniques, and evaluating the efficacy of this type of GUI based scripting method as a training aid to planners/dosimetrists.

## AUTHOR CONTRIBUTIONS

Willem P. E. Boonzaier was responsible for conceptualization and writing of the original draft, study design and methodology, primary investigation and initial formal analysis, data curation, project administration, and software coding. Lourens J. Strauss contributed to writing and co‐authoring sections of the manuscript, study design, supervision, visualization and graphical work, validation, review & editing, and formal analysis.

## CONFLICT OF INTEREST STATEMENT

The authors declare no conflicts of interest.

## References

[1] Manson EN , Hasford F , Trauernicht C , et al. Africa's readiness for artificial intelligence in clinical radiotherapy delivery: medical physicists to lead the way. Phys Medica. 2023;113:102653. 10.1016/j.ejmp.2023.102653 37586146

[2] Jones S , Thompson K , Porter B , et al. Automation and artificial intelligence in radiation therapy treatment planning. J Med Radiat Sci. 2023:1‐9. 10.1002/jmrs.729 PMC11177028 37794690

[3] Fogliata A , Cozzi L , Reggiori G , et al. RapidPlan knowledge based planning: iterative learning process and model ability to steer planning strategies. Radiat Oncol. 2019;14(1):1‐12. 31666094 10.1186/s13014-019-1403-0 PMC6822368

[4] Chung CV , Khan MS , Olanrewaju A , et al. Knowledge‐based planning for fully automated radiation therapy treatment planning of 10 different cancer sites. Radiother Oncol. 2025;202:110609. 39486482 10.1016/j.radonc.2024.110609

[5] Hirashima H , Nakamura M , Miyabe Y , Uto M , Nakamura K , Mizowaki T . Monitoring of mechanical errors and their dosimetric impact throughout the course of non‐coplanar continuous volumetric‐modulated arc therapy. Radiat Oncol. 2018;13(1):1‐8. 29444693 10.1186/s13014-018-0972-7 PMC5813375

[6] Clements M , Schupp N , Tattersall M , Brown A , Larson R . Monaco treatment planning system tools and optimization processes. Med Dosim. 2018;43(2):106‐117. 29573922 10.1016/j.meddos.2018.02.005

[7] Tonneau M , Roos M , Cayez R , et al. Multicriteria optimization of radiation therapy: towards empowerment and standardization of reverse planning for head and neck squamous cell carcinoma. Cancer/Radiotherapie. 2024;28(4):317‐322. 38937203 10.1016/j.canrad.2024.01.003

[8] Cardenas CE , Cardan RA , Harms J , Simiele E , Popple RA . Knowledge‐based planning, multicriteria optimization, and plan scorecards: a winning combination. Radiother Oncol. 2025;202:110598. 39490417 10.1016/j.radonc.2024.110598 PMC11663123

[9] Ayala R , Ruiz G , Valdivielso T . Automatizing a nonscripting TPS for optimizing clinical workflow and reoptimizing IMRT/VMAT plans. Med Dosim. 2019;44(4):409‐414. 30952384 10.1016/j.meddos.2019.02.006

[10] Huang X , Quan H , Zhao B , Zhou W , Chen C , Chen Y . A plan template‐based automation solution using a commercial treatment planning system. J Appl Clin Med Phys. 2020;21(5):13‐25. 10.1002/acm2.12848 PMC7286016 32180351

[11] Naccarato S , Rigo M , Pellegrini R , et al. Automated planning for prostate stereotactic body radiation therapy on the 1.5 T MR‐Linac. Adv Radiat Oncol. 2022;7(3):1‐12. 10.1016/j.adro.2021.100865 PMC8850203 35198836

[12] Li XA , Alber M , Deasy JO , et al. The use and QA of biologically related models for treatment planning report of AAPM Task Group 166. New York. Medical physics. 2012(166):7‐10. 10.1118/1.3685447 22380372

[13] Wu Q , Djajaputra D , Liu HH , Dong L , Mohan R , Wu Y . Dose sculpting with generalized equivalent uniform dose. Med Phys. 2005;32(5):1387‐1396. 15984690 10.1118/1.1897464

[14] Thieke C , Bortfeld T , Niemierko A , Nill S . From physical dose constraints to equivalent uniform dose constraints in inverse radiotherapy planning. Med Phys. 2003;30(9):2332‐2339. 14528955 10.1118/1.1598852

[15] Stavrev P , Hristov D , Warkentin B , Sham E , Stavreva N , Fallone BG . Inverse treatment planning by physically constrained minimization of a biological objective function. Med Phys. 2003;30(11):2948‐2958. 14655942 10.1118/1.1617411

[16] Thomas E , Chapet O , Kessler ML , Lawrence TS , Ten Haken RK . Benefit of using biologic parameters (EUD and NTCP) in IMRT optimization for treatment of intrahepatic tumors. Int J Radiat Oncol Biol Phys. 2005;62(2):571‐578. 15890602 10.1016/j.ijrobp.2005.02.033

[17] Chapet O , Thomas E , Kessler ML , Fraass BA , Ten Haken RK . Esophagus sparing with IMRT in lung tumor irradiation: an EUD‐based optimization technique. Int J Radiat Oncol Biol Phys. 2005;63(1):179‐187. 16111587 10.1016/j.ijrobp.2005.01.028

[18] Spalding AC , Jee KW , Vineberg K , et al. Potential for dose‐escalation and reduction of risk in pancreatic cancer using IMRT optimization with lexicographic ordering and gEUD‐based cost functions. Med Phys. 2007;34(2):521‐529. 17388169 10.1118/1.2426403

[19] Wu Q , Mohan R , Niemierko A , Schmidt‐Ullrich R . Optimization of intensity‐modulated radiotherapy plans based on the equivalent uniform dose. Int J Radiat Oncol Biol Phys. 2002;52(1):224‐235. 11777642 10.1016/s0360-3016(01)02585-8

[20] AAPM Task Group 166 TPC . The Use and QA of Biologically Related Models for Treatment Planning Report of AAPM Task Group 166 (2012).

[21] Ezzell GA , Burmeister JW , Dogan N , et al. IMRT commissioning: multiple institution planning and dosimetry comparisons, a report from AAPM Task Group 119. Med Phys. 2009;36(11):5359‐5373. 19994544 10.1118/1.3238104

[22] Kaviarasu K , Raj N , Murthy K , Babu A , Prasad B . Impact of dose rate on accuracy of intensity modulated radiation therapy plan delivery using the pretreatment portal dosimetry quality assurance and setting up the workflow at hospital levels. J Med Phys. 2015;40(4):226‐232. 26865759 10.4103/0971-6203.170786 PMC4728894

[23] Kruse JJ . On the insensitivity of single field planar dosimetry to IMRT inaccuracies. Med Phys. 2010;37(6):2516‐2524. 20632563 10.1118/1.3425781

[24] IAEA . Technical Reports SeriEs No. 483 Dosimetry of Small Static Fields Used in External Beam Radio therapy (2017).

[25] Kerns JR , Childress N , Kry SF . A multi‐institution evaluation of MLC log files and performance in IMRT delivery. Radiat Oncol. 2014;9(1):1‐10. 25112533 10.1186/1748-717X-9-176 PMC4251954

[26] Menzel HG . Prescribing, recording, and reporting photon‐beam Intensity‐Modulated Radiation Therapy (IMRT). J ICRU. 2010;10(1):1‐106.

[27] Marks LB , Yorke ED , Jackson A , et al. Use of normal tissue complication probability models in the clinic. Int J Radiat Oncol Biol Phys. 2010;76(3 SUPPL.). 10.1016/j.ijrobp.2009.07.1754 PMC4041542 20171502

[28] Niemierko A . Reporting and analyzing dose distributions: a concept of equivalent uniform dose. Med Phys. 1997;24(1):103‐110. 9029544 10.1118/1.598063

[29] Busch K , Andersen AG , Casares‐Magaz O , et al. Evaluating the influence of organ motion during photon vs. proton therapy for locally advanced prostate cancer using biological models. Acta Oncol (Madr). 2017;56(6):839‐845. 10.1080/0284186X.2017.1317107 28464733

[30] Hysing LB , Skorpen TN , Alber M , Fjellsbø LB , Helle SI , Muren LP . Influence of organ motion on conformal vs. intensity‐modulated pelvic radiotherapy for prostate cancer. Int J Radiat Oncol Biol Phys. 2008;71(5):1496‐1503. 18538493 10.1016/j.ijrobp.2008.04.011

[31] Menzel HG . The international commission on radiation units and measurements report 83. J ICRU. 2010;10(1):1‐106. 10.1093/jicru/ndq025 24173704

[32] Nakamura K , Okuhata K , Tamura M , et al. An updating approach for knowledge‐based planning models to improve plan quality and variability in volumetric‐modulated arc therapy for prostate cancer. J Appl Clin Med Phys. 2021;22(9):113‐122. 34338435 10.1002/acm2.13353 PMC8425874

